# Reprogrammed mesenchymal stem cells derived from iPSCs promote bone repair in steroid-associated osteonecrosis of the femoral head

**DOI:** 10.1186/s13287-021-02249-1

**Published:** 2021-03-12

**Authors:** Meiling Zhou, Jiaoya Xi, Yaofeng Cheng, Denglong Sun, Peng Shu, Shuiqing Chi, Shuo Tian, Shunan Ye

**Affiliations:** 1 Department of Physiology, School of Basic Medicine, Tongji Medical College, Huazhong University of Science and Technology, Wuhan, Hubei China; 2 Department of Orthopedics, Union Hospital, Tongji Medical College, Huazhong University of Science and Technology, Wuhan, 430022 Hubei China; 3 Department of Orthopedics, Suizhou Central Hospital, Hubei University of Medicine, Suizhou, Hubei China; 4 Tongji Medical College, Huazhong University of Science and Technology, Wuhan, Hubei China

**Keywords:** Human induced pluripotent stem cells, Osteonecrosis of the femoral head, Mesenchymal stem cell, Cell therapy, Bone regeneration

## Abstract

**Background:**

Cellular therapy based on mesenchymal stem cells (MSCs) is a promising novel therapeutic strategy for the osteonecrosis of the femoral head (ONFH), which is gradually becoming popular, particularly for early-stage ONFH. Nonetheless, the MSC-based therapy is challenging due to certain limitations, such as limited self-renewal capability of cells, availability of donor MSCs, and the costs involved in donor screening. As an alternative approach, MSCs derived from induced pluripotent stem cells (iPSCs), which may lead to further standardized-cell preparations.

**Methods:**

In the present study, the bone marrow samples of patients with ONFH (*n* = 16) and patients with the fracture of the femoral neck (*n* = 12) were obtained during operation. The bone marrow-derived MSCs (BMSCs) were isolated by density gradient centrifugation. BMSCs of ONFH patients (ONFH-BMSCs) were reprogrammed to iPSCs, following which the iPSCs were differentiated into MSCs (iPSC-MSCs). Forty adult male rats were randomly divided into following groups (*n* = 10 per group): (a) normal control group, (b) methylprednisolone (MPS) group, (c) MPS + BMSCs treated group, and (d) MPS + iPSC-MSC-treated group. Eight weeks after the establishment of the ONFH model, rats in BMSC-treated group and iPSC-MSC-treated group were implanted with BMSCs and iPSC-MSCs through intrabone marrow injection. Bone repair of the femoral head necrosis area was analyzed after MSC transplantation.

**Results:**

The morphology, immunophenotype, in vitro differentiation potential, and DNA methylation patterns of iPSC-MSCs were similar to those of normal BMSCs, while the proliferation of iPSC-MSCs was higher and no tumorigenic ability was exhibited. Furthermore, comparing the effectiveness of iPSC-MSCs and the normal BMSCs in an ONFH rat model revealed that the iPSC-MSCs was equivalent to normal BMSCs in preventing bone loss and promoting bone repair in the necrosis region of the femoral head.

**Conclusion:**

Reprogramming can reverse the abnormal proliferation, differentiation, and DNA methylation patterns of ONFH-BMSCs. Transplantation of iPSC-MSCs could effectively promote bone repair and angiogenesis in the necrosis area of the femoral head.

**Supplementary Information:**

The online version contains supplementary material available at 10.1186/s13287-021-02249-1.

## Background

Osteonecrosis of the femoral head (ONFH) is a progressive and refractory disease that is common in orthopedics. Although ONFH is not fatal, it is persistently painful, seriously affecting the quality of life of the patients. The usual cause of ONFH is the inadequate blood supply to the trabecular bone in the femoral head, ultimately leading to bone cell death due to various factors, such as excessive alcohol consumption, hormone application, trauma, and diseases of connective tissue, which is followed by the collapse of the articular cartilage and subsequent osteoarthritis [1–6]. According to a nationally representative survey conducted from June 2012 to August 2013, the number of non-traumatic ONFH cases in the Chinese population aged 15 years and above was 8.12 million [7]. ONFH is becoming a significant public health challenge in China as well as globally. If timely treatment is not received, ONFH would lead to the collapse of the femoral head, which requires total hip arthroplasty (THA) [8]. However, the onset of ONFH at a younger age renders the long-term outcome of THA unpredictable for such cases [9]. Therefore, better treatment approaches for preventing femoral head collapse are required. Currently, the pathogenesis of ONFH is not clearly understood, although certain recent studies have demonstrated a strong association between ONFH and the reduction or alterations of BMSCs or other progenitor cells in the proximal femur, and it is gradually accepted that ONFH originates at the cellular level [10–15].

BMSCs are a kind of adult stem cells, which exhibit the ability of self-renewal and multilineage differentiation under specific conditions [16, 17]. BMSCs play an important role in bone metabolism homeostasis, which has a close association with the balance of osteogenic and adipogenic differentiation [18, 19]. Recent studies have demonstrated that the number of BMSCs and their activity are decreased in ONFH patients [2, 19, 20]. The past few years have witnessed an extensive application of stem cell transplantation in several ischemic diseases, including myocardial infarction, stroke, and retinal degeneration. Autologous bone marrow transplantation for ONFH treatment was first reported by Hernigou et al. [21], who demonstrated that the patients having a higher number of progenitor cells transplanted into their hips, presented better outcomes. With an increase in the number of clinical research works being reported in this field, mesenchymal stem cells (MSCs) transplantation is gradually becoming the most preferred alternative option for the treatment of ONFH [22–25]. Nonetheless, this approach has certain potential limitations. First, obtaining an adequate number of MSCs, which is important as the outcomes are linked to the number of transplanted cells, is difficult in the case of certain patients. Second, although there is little change in the frequency of autologous MSCs with age, unfortunately, the biological activity of MSCs varies significantly among individuals, with the elderly patients or the patients with systemic diseases usually having MSCs with a lower self-renewal ability or an impaired function [26–28]. Third, the proliferation potential of MSCs is limited, and certain important biological functions might change with the extensive expansion of the culture in vitro [29]. Therefore, it is necessary to explore and establish an alternate standardized and inexhaustible source of MSCs exhibiting high activity for cellular therapy.

Stem cell-derived MSCs have emerged as an important alternative source for BMSCs, providing a feasible solution to the above-stated problems. Previously, human embryonic stem cells (ESCs) were commonly used as a source of MSCs. However, the extensive application of ESC-MSCs in the clinical intervention was limited greatly due to concerns related to ethical issues and immunogenicity. A potentially inexhaustible cell resource with a pluripotency potential similar to the ESCs is represented by the induced pluripotent stem cells (iPSCs) generated from autologous somatic cells. The iPSCs present the potential for patient-specific autologous therapies, thereby eliminating most of the problems associated with immune incompatibility and ethical issues, and introducing a novel direction for regenerative medicine based on cell transplantation [30]. In addition, the use of iPSCs would remove one of the main obstacles in the path of clinical cell therapy, i.e., the shortage of functional autologous MSCs. In recent years, MSCs derived from iPSCs (iPSC-MSCs) have provided breakthrough progress in the treatment of immune system-related diseases [31], myocardial infarction [32], and bone defects [33]. However, reports on ONFH treatment are scarce. Our study is to investigate the possibility of iPSC-MSCs in the treatment of early-stage steroid-induced ONFH. The present study demonstrated that it was possible to establish a patient-specific iPSC cell line, which would provide a stable and reliable source of iPSC-derived MSCs for cellular therapy in ONFH. Moreover, iPSC-MSCs are equivalent to normal BMSCs in the treatment of ONFH.

## Methods

### Patients’ information

With the approval of the ethics committee, as stated previously, a total of 28 bone marrow donors were recruited in the present study. The recruited participants were the patients who underwent total hip replacement between May 2016 and November 2017, at the Wuhan Union Hospital, Huazhong University of Science and Technology. Among these 28 patients, 16 were diagnosed with ONFH based on the medical imaging and histopathology results. The bone marrow samples (10 mL each) for the experiments were obtained during surgery. The bone marrow samples from the 12 patients with fractured femoral neck served as the source of normal control cells. The clinical characteristics of the participating patients are summarized in Supporting Information Table S1.

### Isolation, culture, and identification of human BMSCs

The BMSCs derived from the ONFH patients (ONFH-BMSCs) and the control BMSCs were isolated and cultured, as reported previously [34]. Briefly, mononuclear cells were separated from the bone marrow samples by an equal volume 1.073 g/mL of Percoll solution (Sigma, USA) at 2000 rpm for 25 min and resuspended in the DMEM/F12 medium (HyClone, USA) containing 10% fetal bovine serum (Gibco, USA) and 1× penicillin/streptomycin. The cells were seeded in 60-mm culture dishes (Corning, USA) at a density of 10^5^ cells/cm^2^ and incubated at 37 °C with 5% CO_2_. The non-adherent cells were removed after 3 days of culture, and the medium was changed every 2 days. When 80–90% confluence was reached, the cells were harvested using 0.25% trypsin containing 0.02% EDTA (Invitrogen, USA), and were reseeded at the ratio of 1:3 for initial subculture. After three passages, the BMSCs were ready for the subsequent experiments.

The immunophenotypic study of the BMSCs was performed by fluorescence-activated cell sorting (FACS). Briefly, the cells were suspended in phosphate-buffered saline (PBS) at a concentration of 10^6^ cells/mL. The following antibodies were used in the analysis: mouse anti-human CD44-PE, CD13-PE, CD73-APC, CD34-PE, CD105-FITC, and HLA-DR-FITC (BioLegend, CA). The cells were analyzed under the Flow Cytometry Caliber instrument (BD Biosciences, LSR II, USA). BD Diva software version 6.1.3 was used for data collection and analysis (Supporting Information Figure S1).

### Generation iPSCs from ONFH-BMSCs

Retroviruses were produced as described previously [35]. Briefly, 293 T cells (a gift from Prof. Bin Zhang of Huazhong University of Science and Technology) were plated at 80% confluence per 100 mm dish, each pMXs vector encoding the human transcription factors OCT3/4 (Addgene, 22684), SOX2 (Addgene, 115752), KLF4 (Addgene, 116659), and c-MYC (Addgene, 116661) was transduced into 293 T cells with PCL-Ampho packaging plasmids using calcium phosphate transfection. The virus-containing supernatants were filtered through a 0.45 μm pore-size filter (Millipore, USA) and centrifuged at 4000 g for 25 min with Amicon Ultra (Millipore, USA) to concentrate. ONFH-BMSCs (passage3) seeded at a density of 3× 10^4^ cells/well in a 6-well plate and cultured for 24 h. After starved with DMEM medium for 2 h, cells were transduced with equal amounts of four concentrated viruses supplemented with 8 μg/ml polybrene (Sigma, USA). The day was considered as day 0. Transduction was repeated after 2 days. On day 6, cells were harvested with 0.25% trypsin-EDTA, and 5 × 10^4^ cells were seeded on mouse embryonic fibroblasts (MEFs) feeder in a gelatin-coated 100 mm culture dish cultured with mTeSR-1 medium (Stemcell Technologies, Canada). The medium was changed every other day. ES-like colonies usually appeared from day 13. About on day 20–22, the ES-like colonies were mechanically isolated. Every single picked colony was cultured and expanded in a Matrigel-coated 24-well plate with the mTeSR-1 medium. The cells generated from an individual colony were defined as the individual iPS cell line and used for further experiments.

### Alkaline phosphatase (AP) staining

The iPSCs were fixed with 4% paraformaldehyde for 15 min at room temperature. Then, cells were stained with Naphthol phosphate (0.2 mg/ml; Sigma) in Fast Red (1 mg/ml; Sigma) that was prepared in Tris-HCl (pH 9.2) for 30 min in the dark at room temperature. Images were taken randomly by a microscope (Olympus, IX71, Japan).

### Teratoma formation and karyotype analysis of iPSCs

To form teratomas, the iPSCs were harvested by 0.5 mM EDTA, 10^7^ cells resuspended in 100 μl DMEM/F12, and 200 μl of Matrigel. Cells were injected into the dorsal flank of a SCID mouse. Ten weeks after injection, tumors were collected and fixed with 4% paraformaldehyde. Subsequently, paraffin-embedded tumors were sectioned and stained with hematoxylin and eosin.

Karyotype analyses were performed in Cytogenetic Laboratory, Huazhong University of Science and Technology. In brief, the cells were treated with 10 mg/ml colchicine for 60 min at 37 °C, harvested with 0.5 mM EDTA, exposed to 0.075 M KCl hypotonic solution for 30 min, and fixed five times with methanol/acetic acid (3:1). The cells were then analyzed by Giemsa-banding.

### Derivation of MSCs from iPSCs

The differentiation of the iPSCs into MSCs was initiated with the formation of embryoid bodies (EBs), as reported previously [36] with certain modifications. Briefly, the iPSCs were seeded at a density of 2 × 10^4^ cells/cm^2^ in non-adhesive culture dishes containing the mTeSR-1 medium supplemented with ROCK inhibitor Y27632 (10 μM; Selleck, USA), which was added to promote the formation of EBs on the first day of the suspension culture. On day 2, the medium was replaced with 20% knockout serum replacer medium (Gibco, USA). After 1 week of suspension culture, the EBs were transferred to a standard culture dish and were cultured in MSC complete culture medium. When 90% confluence was reached, the differentiating cells were passaged at the ratio of 1:3, and the process was continued until the cells exhibited MSC-like morphology and analyzed for expression of MSC surface markers by flow cytometry weekly. After approximately six passages, the cells exhibited a typical fibroblast-like growth pattern and expressed MSC surface markers, and at this stage, the cells were referred to as the iPSC-MSCs (passage 1).

### Osteogenic and adipogenic differentiation of MSCs

Osteogenic differentiation was induced with DMEM-L medium (HyClone, USA) supplemented with 10% FBS, 10 mmol/l β-glycerophosphate (Sigma, USA), 0.1 μM dexamethasone (Sigma, USA), and 50 μg/ml ascorbic acid (Sigma, USA). The medium was changed every 3 days. After 2 weeks, osteogenic differentiation was analyzed by Alizarin Red staining. Briefly, cells were fixed with 4% paraformaldehyde for 15 min, then stained with 40 mM Alizarin Red solution (pH 4.2) (Aladdin, China) for 10 min at room temperature, and washed with PBS until nonspecific staining. Images were taken randomly using a microscope. The quantitative analysis was performed using 10% cetylpyridinium chloride (Sigma, USA) extraction. Absorbance was measured at 560 nm. Experiments were repeated six times.

For adipogenic differentiation, cells were cultured in DMEM-H medium (HyClone, USA) supplemented with 10% FBS, 0.5 mM isobutylmethylxanthine (Sigma, USA), 1 μM dexamethasone, and 10 μg/ml insulin [37]. After 2 weeks, cells were stained with Oil Red O solution (Sigma-Aldrich, USA). Briefly, cells were fixed with 4% paraformaldehyde, stained for 10 min at room temperature with filtered oil red O solution. After washed twice with PBS, cells were counterstained with hematoxylin for 5 min. For quantitative analysis, cells were treated with isopropanol and shook for 15 min at room temperature. After completely dissolved, the absorbance was measured at 490 nm [38]. Experiments were repeated five or six times.

### Quantitative real-time PCR

Total RNA of cells was extracted using Trizol reagent (Invitrogen) through standard protocols according to the instructions of the manufacturer. The concentration and quality of total RNA were checked by NanoDrop 2000 (Thermo Fisher Scientific) and gel electrophoresis. Total RNA was reversely transcribed into cDNA. Real-time PCR was performed in the CFX Connect™ Real-Time PCR Detection System (Bio-Rad) using SYBR Premix Ex Taq (TOYOBO). The relative expression of mRNA was quantified using the ΔΔCt method [39]. The primers used for amplification are listed in Supplemental Table S2.

### Cell proliferation assay

Cell viability was detected at different times by cell counting kit-8 (Dojindo) according to the manufacturer’s instructions. Cells were seeded in the 96-well plate at a density of 8000 cells/well. The absorbance was measured at 450 nm, and the corresponding cell number was calculated according to the standard curve. Six to eight independent experiments were performed. BrdU labeling was performed to assess cell proliferation. 10^4^ cells were seeded in a 24-well-plate supplemented with 10 μM 5-Bromo-2-deoxyuridine (BrdU; Sigma, USA) and cultured for 48 h. After fixation with 4% paraformaldehyde, BrdU incorporation was detected by fluorescent staining using an anti-BrdU antibody, and nuclei were stained with DAPI. The total number of cells was determined by counting DAPI-positive cells. Images were taken randomly by a fluorescence microscope (Olympus, IX71, Japan). The percentage of proliferating cells was determined by dividing the number of BrdU/DAPI double-positive cells by the total number of cells. The proliferation assays were performed by more than 4 independent experiments, and each experiment randomly selected 4 images for statistics.

### Senescence-associated β-galactosidase staining

In order to study the senescence of the BMSCs (Passage 4) and iPSC-MSCs (Passage 4), the Senescence Cells Histochemical Staining Kit (Beyotime Biotechnology, China) was used in accordance with the manufacturer’s instructions. Random images were photographed during observation under a microscope (Olympus IX71, Japan). The number of cells with intracellular blue deposits divided by the total number of cells was the ratio of senescent cells. Five independent experiments were performed to ensure reproducibility.

### DNA methylation profiling

Genomic DNA was isolated with the DNA Blood Midi Kit (Tiangen, China). DNA quality was assessed using a spectrophotometer and gel electrophoresis. The DNA samples (500 ng) were bisulfite converted using the EZ DNA Methylation Kit (Zymo, Irvine, CA) in accordance with the manufacturer’s instructions. The DNA methylation profiles were analyzed using Infinium HumanMethylation850 BeadChip (Illumina) in accordance with the manufacturer’s instructions. The initial analysis was performed in Genomestudio 2010.3 (Modul M Version 1.8.5) at the Shanghai Biotechnology Corporation (Shanghai, China). The obtained data were normalized with the internal controls according to Illumina’s standard procedures. The level of methylation at each locus was calculated using the GenomeStudio Methylation module as the *β* value (ranging from 0 to 1). The CpG sites with *β* value < 0.2 and > 0.8 were regarded as hypomethylated and hypermethylated sites, respectively. The number of beads per feature varying between chips and the *β* values was calculated as the average of at least three experimental replica values.

### In vivo animal experiment

A total of 40 adult male Sprague Dawley rats were used for the study. The experiments were performed in the Animal Experiment Center of Tongji Medical College, Huazhong University of Science and Technology. All procedures were conducted in strict accordance with the guidelines for the Care and Use of Laboratory Animals.

### Steroid-induced ONFH rat model

A total of 40 male Sprague Dawley rats (8–10 weeks old and weighing 250–300 g) were purchased from the Animal Experiment Center of Tongji Medical College, Huazhong University of Science and Technology. The rats were assigned randomly to the following groups (*n* = 10 per group): (a) normal control group, (b) methylprednisolone (MPS) group, (c) MPS + BMSC-treated group, and (d) MPS + iPSC-MSCs-treated group. ONFH was induced in the relevant groups of rats using MPS, as described previously [40, 41] with slight modifications. Briefly, the rats were injected intraperitoneally (i.p.) with a 20 μg/kg dose of lipopolysaccharide (LPS; Sigma, USA), once a day for three consecutive days. Subsequently, the rats were intramuscularly (i.m.) injected with MPS (Pfizer Pharmaceutical, China) at a dose of 40 mg/kg, once a day for three consecutive days. Thereafter, all the rats were carefully housed and maintained for the next 8 weeks.

### Cell labeling and implantation

Cell labeling and transplantation were performed as previously described [15, 40, 42]. Briefly, when the cells reached a confluence of 50–60%, BrdU was added at a final concentration of 10 μM to label the MSCs. After approximately 90% confluence was reached, the cells were collected and preserved on ice until transplantation. Before transplantation, a sample of cells was collected for the evaluation of the effectiveness of cell labeling. Eight weeks after the establishment of the ONFH model, intrabone marrow injection was performed as previously described [40, 42]. Briefly, the animals were anesthetized, and the lower limb was disinfected. An incision was made in the center of the knee to open the knee cavity, and a 2-ml needle was inserted into the intercondylar, penetrating the marrow cavity of the femur and approaching the proximal femur. Then, 0.2 ml of BMSCs (10^7^ cells/ml) or iPSC-MSCs (10^7^ cells/ml) was injected. After injection, the needle was left in the tissue for 1 min before slowly withdrawing, and the marrow cavity was sealed with sterilized bone wax. Animals returned to their homes for standard husbandry. The rats of the BMSC-treated group, and the iPSC-MSC-treated group were injected with BMSCs and iPSC-MSCs, respectively. The MPS group rats were injected with 0.2 ml PBS. The duration of the intervention was 8 weeks. The femoral head samples were obtained after the rats were sacrificed ethically.

### Micro-CT analysis

Micro-CT analysis was performed at week 4 and week 8 after cell implantation, using SkyScan 1176 scanner (Bruker MicroCT, Kontich, Belgium). The femoral heads were fixed with 4% paraformaldehyde and subsequently analyzed under SkyScan1176 scanner. The scanner was set at a resolution of 9 μm per pixel. The images were reconstructed and analyzed using the NRecon software (Version 1.5.1.4, Skyscan). The region of interest (ROI) for the analysis and comparison of the trabecular parameters was determined based on a previous study [43]. The trabecular bone parameters analyzed and quantified to determine the relative amount of the bone within the femoral head were bone mineral density (BMD), bone volume (BV), bone volume/total volume (BV/TV), bone surface/total volume (BS/TV), trabecular separation (Tb.Sp), trabecular thickness (Tb.Th), and trabecular number (Tb.N).

### Immunofluorescence and histological analysis

To perform the immunofluorescence analysis, the cells were fixed with 4% paraformaldehyde for 15 min, followed by washing with PBS and subsequent treatment with 5% bovine serum albumin (BSA, Biosharp) in PBS containing 0.1% Triton X-100 for 1 h. The primary antibodies used in the experiment were rabbit anti-human OCT4 (1:150; Cell Signaling Technology, USA), rabbit anti-human SOX2 (1:150; Cell Signaling Technology, USA), rabbit anti-human Nanog (1:150; Cell Signaling Technology, USA), mouse anti-human TRA1–81 (1:150; Cell Signaling Technology, USA), TRA1–60 (1:150; Cell Signaling Technology, USA), SSEA4 (1:200; Abcam, UK), mouse anti-CD31(1:100; Abcam, UK), mouse anti-VEGF (1:100; Abcam, UK), and mouse anti-BrdU (1:100; Proteintech, China). The secondary antibodies used were as follows: TRITC-conjugated goat anti-Mouse-IgG (1:100; Abclonal, China), FITC-conjugated goat anti-Mouse-IgG (1:100; Abclonal, China), TRITC-conjugated goat anti-rabbit IgG (1:100; Abclonal, China), and HRP-conjugated goat anti-Mouse-IgG (1:100; Proteintech, China). Cell nuclei were stained with DAPI (Sigma, USA). Images were acquired during observation under a fluorescence microscope (Olympus, IX71, Japan).

The harvested femoral head tissues were fixed with 4% paraformaldehyde, decalcified using 10% EDTA solution (pH 7.4), dehydrated, and finally embedded in paraffin. The paraffin-embedded sections were then stained with hematoxylin-eosin (HE, Goodbio) as previously reported [44]. For Masson staining, the paraffin-embedded section was stained according to the manufacturer’s instructions. All the stained sections were observed under a microscope (Olympus IX71, Japan).

### Statistical analysis

Statistical analysis charting was performed in GraphPad Prism 7.0 software (GraphPad, San Diego, CA). All results were presented as mean ± SEM unless otherwise stated. Independent-sample *t* tests were used to compare the means of two different groups. One way-ANOVA was performed for multiple comparisons, with *p* < 0.05 as the threshold of statistical significance. All experiments were repeated three times or more.

## Results

### Induction of iPSCs from ONFH-BMSCs

**Fig. 1:**
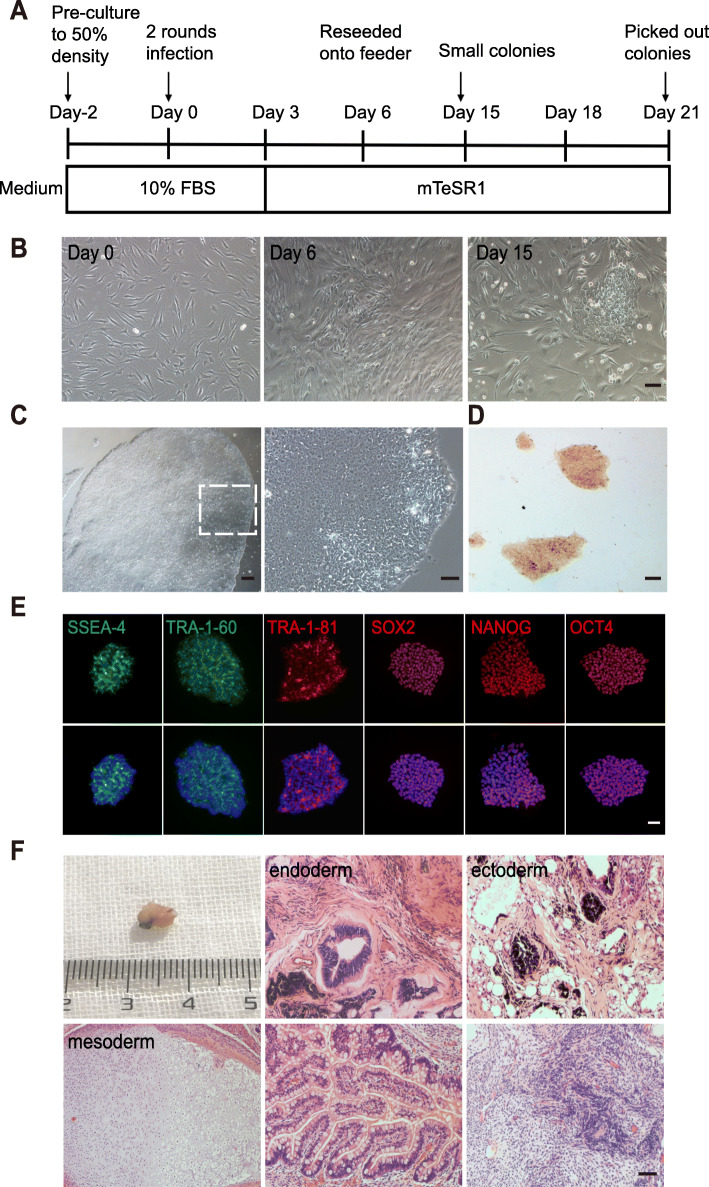
Induction and characterization of iPSCs from ONFH-BMSCs. **a** Timeline for the reprogramming process. **b** Cell morphology of ONFH-BMSCs during reprogramming at different times. **c** Typical image of hES cell-like colony on matrigel (left) and image at high magnification (right). **d** Alkaline phosphatase-positive colonies. **e** Immunofluorescence staining of the pluripotency markers. **f** Teratoma in vivo, which contains all three embryonic germ layers such as respiratory epithelium (upper middle) and gland (lower middle); cartilage (lower left); pigmentary epithelium (upper right); and neural epithelium (lower right). Scale bar indicated 100 μm in (**b**) and (**d**), **c** 200 μm, and **e**, **f** 50 μm. D day

MSCs were isolated from the bone marrow samples of the ONFH patients (ONFH-BMSCs), and the culture was expanded for three passages. As depicted in Figure S1, the results of the flow cytometric analysis (FACS) revealed a high expression of MSC markers CD44 (99.3%), CD13 (99.2%), CD73 (99.4%), and CD105 (96.8%), and almost no expression of CD34 (0.1%) and HLA-DR (0.5%). The timeline of the reprogramming process is summarized in Fig. 1a. At passage three, the cells were infected with retroviruses encoding the human transcription factors OCT4, SOX2, KLF4, and c-MYC. The cells exhibiting mesenchymal-epithelial transformation (MET) appeared on day 6 after transfection, while small colonies emerged approximately at day 15 (Fig. 1b). The colonies exhibiting distinct flat and tight ESC-like morphology with a clear boundary and a high nucleus-to-cytoplasm ratio were selected and expanded further for validation (Fig. 1c). These clones exhibited alkaline phosphatase activity (Fig. 1d) and expressed endogenous pluripotency markers Oct4, Sox2, Nanog, TRA-1–60, TRA-1–81, and SSEA-4 (Fig. 1e). To identify the pluripotency in vivo, the cells were injected in the SCID mice to observe the teratoma formation, and a standard teratoma containing all the three embryonic germ layers could be observed (Fig. 1f). The karyotype analysis revealed that these cells retained a normal karyotype (Figure S2). Collectively, these results indicated a successful generation of iPSCs from ONFH-BMSCs.

### MSCs derived from iPSCs exhibited similar immunophenotype and differentiation potential to cultured normal BMSCs

**Fig. 2:**
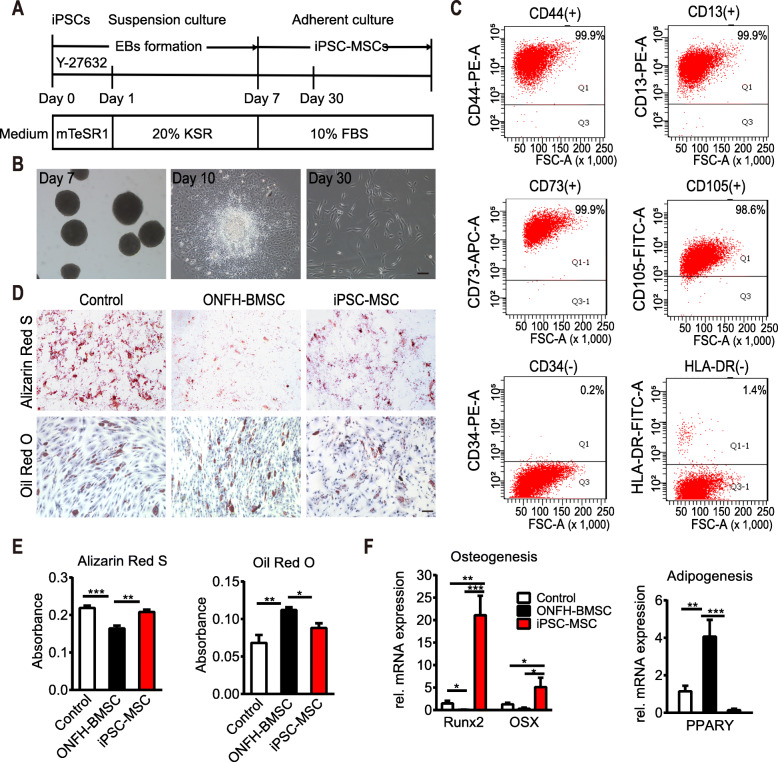
Redifferentiation and characterization of iPSC-MSCs. **a** Timeline for the generation of iPSC-MSCs. **b** Phase contrast images of iPSC-MSCs during differentiation with EB formation. **c** Flow cytometry analysis of iPSC-MSCs. **d** Differentiation potential toward osteogenic and adipogenic lineage, adipogenesis detected by Oil Red O, and osteogenesis by Alizarin Red staining at day 14 after differentiation at passage 4. **e** The quantitative analysis of Oil red O (control vs iPSC-MSC group, *p* = 0.146, *n* = 5 per group) and Alizarin Red staining (control vs iPSC-MSC group, *p* = 0.262, *n* = 6 per group). **f** Gene expression of osteogenic gene Runx2 and OSX and adipogenic gene PPARY after 2 weeks of osteogenic and adipogenic induction at passage 4. For each gene, expression levels were normalized to the control group, *n* = 5 per group. All data are presented as mean ± SEM. One-way ANOVA with Tukey’s adjustment (*p < 0.05; **p < 0.01; ***p < 0.0001). Scale bar indicated 100 μm in all panels

After the establishment of the iPS cell line, the iPSCs were differentiated into MSCs through induction of embryoid body (EB) formation (Fig. 2a). After 7 days of suspension culture, the formed EBs were transferred to a standard culture dish containing MSC complete culture medium. After approximately 30 days, the cells exhibited a typical fibroblast-like morphology (Fig. 2b). FACS results revealed that the majority of these MSC-like cells expressed positive MSC markers (> 98% for CD44, CD13, CD105, and CD73), while the expression of the negative MSC markers, including HLA-DR and CD34, was almost nil (< 2%; Fig. 2c).

The International Society for Cell Therapy regards the in vitro ability of tri-lineage differentiation as one of the main criteria for defining MSCs [45]. Therefore, the capability of osteogenesis and adipogenesis was assessed in the iPSC-MSCs, ONFH-BMSCs, and control BMSCs. After 2 weeks of osteogenic induction, the cells were subjected to alizarin red staining and quantification of calcium content. The results revealed relatively less scattered calcium nodules in the ONFH-BMSC group when compared to the control group (*p* < 0.0001), which was consistent with a previous report [11]. Moreover, the number of calcium nodules in the iPSC-MSC group was significantly higher than that in the ONFH-BMSC group (*p* < 0.01) and similar to that in the control group (*p* = 0.1459, *n* = 6 per group; Fig. 2d, e). To assess adipogenic differentiation, oil red O staining and quantitative analysis were performed after 2 weeks of induction, and the results revealed that the number of lipid vacuoles in the ONFH-BMSC group was significantly higher than that in the control group (*p* < 0.01). Moreover, after the reprogramming, the number of lipid vacuoles in the iPSC-MSC group was significantly lower than that in the ONFH-BMSC group (*p* < 0.01, *n* = 5 per group) and equivalent to that in the control group (*p* = 0.2833; Fig. 2d, e). These findings regarding the in vitro differentiation potential were further verified by up-regulating lineage-specific marker genes (Fig. 2f). Collectively, these results indicated that the generated iPSC-MSCs exhibited the similar immunophenotype and differentiation ability to normal BMSCs and that reprogramming could reverse the differentiation ability of ONFH-BMSCs.

### The generated iPSC-MSCs exhibited faster proliferative capability, immunoregulatory function, and low immunogenicity

**Fig. 3:**
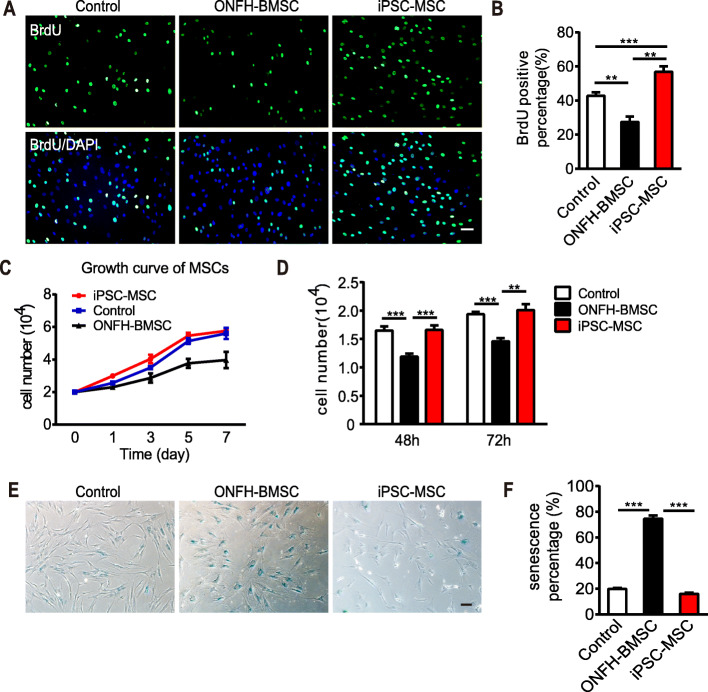
Cell proliferation potential and senescence assay. **a** Fluorescence imaging for BrdU and DAPI labeling of three groups of cells. **b** The quantitative analysis of BrdU-positive percentage of three groups of cells (*n* = 4 per group). **c** Cell growth curve (*n* = 3 per group). **d** CCK8 detection of cell proliferation at different time (control and ONFH-BMSC group, *n* = 6, iPSC-MSC group *n* = 8, control vs iPSC-MSC, *p* > 0.05). **e** β-galactosidase staining for senescence assay. **f** The quantitative analysis of β-galactosidase staining (*n* = 5). All data are shown as mean ± SEM. Statistical differences were calculated using one-way ANOVA with Tukey’s adjustment (**p < 0.01; ***p < 0.0001). Scale bar indicated 50 μm in all panels

The iPSC-MSCs represent a promising substitute for BMSCs or the MSCs derived from adult tissues other than BM. One of the advantages of using iPSC-MSCs is its high self-renewal ability. Therefore, in the present study, the proliferation potential of the generated iPSC-MSCs was assessed using BrdU labeling. The result revealed that ONFH-BMSCs exhibited a significantly decreased proliferation rate compared to control BMSCs (27.52% ± 3.21% vs. 42.84% ± 2.04%, *n* = 4, *p* < 0.01), while a higher proliferation rate was observed for iPSC-MSCs compared to ONFH-BMSCs (56.90% ± 3.16% vs. 27.52% ± 3.21%, *n* = 4, *p* < 0.0001; Fig. 3a, b). The faster proliferation potential of the iPSC-MSCs was further validated by CCK8 assay (*n* = 8; Fig. 3c, d). A long-term in vitro culture is accompanied by an increase in the number of senescent cells and upregulation in the activity of senescence-associated β-galactosidase (SA-β-gal), which is detectable using β-galactosidase staining. The β-galactosidase staining results revealed an accumulation of senescent cells (blue) in the ONFH-BMSC group, and the percentage of the senescent cells was significantly higher than that in the control group (*p* < 0.0001). After reprogramming, the percentage of senescent cells in the iPSC-MSC group was lower compared to both ONFH-BMSCs group (16.01% ± 0.96% vs. 74.64% ± 2.47%, *n* = 5, *p* < 0.0001) and control group (16.01% ± 0.96% vs. 19.79% ± 0.85%, n = 5, *p* < 0.05; Fig. 3e, f).

Currently, MSCs are the most preferred cells for cell replacement therapy. One of the main advantages of MSCs is their low immunogenicity and a certain capacity for immunoregulation. Therefore, MSC transplantation can reduce immunological rejection to a certain extent. In this context, immunogenicity and the immunoregulatory function of the generated iPSC-MSCs were assessed based on the detection of immune-related surface molecules (CD80 and CD86) and co-culture with rat lymphocytes. Costimulatory molecules CD80/CD86 play an important role in the activation, proliferation, and immune response of T lymphocytes. FACS results revealed almost nil expression of CD80 and CD86 (< 1%) in the iPSC-MSCs, similar to BMSCs (Figure S3a, b). It was observed that BMSCs could significantly promote lymphocyte proliferation under PHA stimulation (*p* < 0.0001), while the iPSC-MSCs could inhibit PHA-stimulated lymphocyte proliferation, which indicated that the iPSC-MSCs possessed certain immunomodulatory functions (Figure S3c). The results revealed that the iPSC-MSCs possessed immunoregulatory function and exhibited low immunogenicity. Furthermore, the iPSC-MSCs were injected into SCID mice (*n* = 3), and no tumor formation was observed in these SCID mice even after 3 months of inoculation with iPSC-MSCs. Collectively, these findings indicated that the iPSC-MSCs exhibited faster proliferative capability, immunoregulatory function, and low immunogenicity, without tumor formation.

### DNA methylation profiles of iPSC-MSCs resembled those of normal BMSCs

**Fig. 4:**
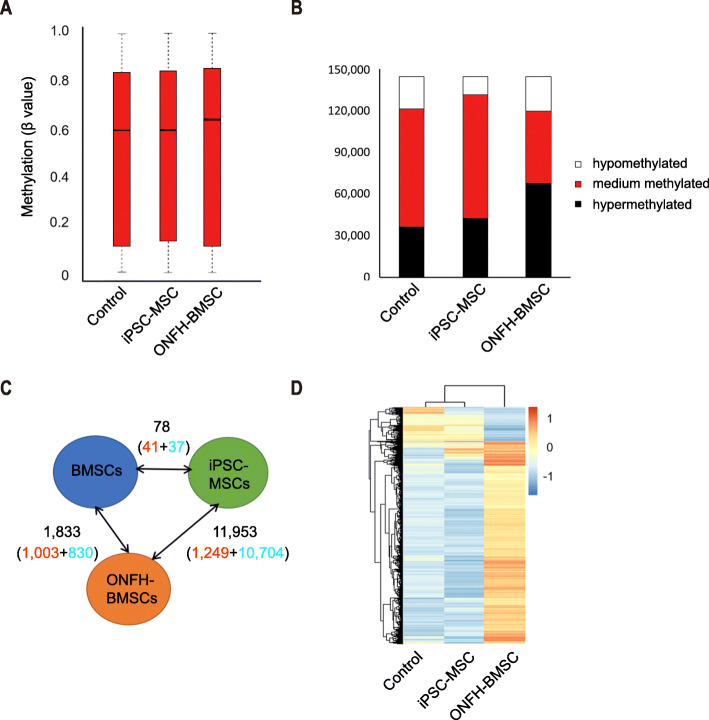
DNA methylation analysis. **a** The median level of all CpG sites (black line represents median). **b** Statistics of CpG sites with different methylation status. **c** Number of CpGs with differential DNAm between normal BMSCs, ONFH-BMSCs and iPSC-MSCs (*p* < 0.05); hypermethylated CpGs (red), hypomethylationare CpGs (blue). **d** Heatmap analysis of 13,432 CpG sites with differential DNAm in normal BMSCs, ONFH-BMSCs, and iPSC-MSCs demonstrated that ONFH-associated DNAm pattern is erased upon reprogramming and not reestablished in iPSC-MSCs. P passage; D donor

Changes in DNA methylation patterns are reported to be associated with the pathogenesis of ONFH [38, 46]. Genome-wide DNA methylation profiling was performed using the Infinium Human Methylation 850 K Bead Chip. The median methylation levels of all the CpG sites in control BMSCs, iPSC-MSCs, and ONFH-BMSCs were approximately 58.8%, 59.5%, and 63.0%, respectively (Fig. 4a). There were 1833 CpG sites exhibiting differential methylation between ONFH-BMSCs and normal BMSCs. In comparison, 11,953 CpG sites exhibited differential methylation upon reprogramming of ONFH-BMSCs into iPSC-MSCs, which contained 10,704 demethylated CpG sites and 1249 hypermethylated CpG sites. Only 78 CpG sites presented differential methylation in iPSC-MSCs versus normal BMSCs (*p* value < 0.05) (Fig. 4b). Hierarchical cluster analysis of the 11,953 CpG sites with differential DNA methylation revealed that the aberrant methylation patterns in ONFH-BMSCs were eliminated with the reprogramming and did not reestablish upon differentiation into iPSC-MSCs. Furthermore, the DNA methylation patterns of iPSC-MSCs were similar to those of the normal BMSCs (Fig. 4c, d). Collectively, these results suggested that reprogramming could reverse the abnormal DNA methylation patterns of ONFH-BMSCs.

### Cellular therapy with iPSC-MSCs prevented bone loss and promoted bone repair in steroid-induced ONFH rat model

The therapeutic effects of iPSC-MSCs were investigated by transplanting these cells into the adult rat ONFH model. Before transplantation, the cells were labeled with BrdU to facilitate tracking of their post-transplantation fate and distribution. ONFH was induced in the rats using methylprednisolone (MPS), as described previously [15, 43]. The rats were randomly divided into the following four groups (*n* = 10 per group): normal BMSCs implant group (MPS + BMSCs), iPSC-MSCs implant group (MPS + iPSC-MSCs), MPS group (MPS + PBS), and normal control group. The intramedullary injection was performed 8 weeks after the induction of the ONFH model. Observation upon 4 weeks after the intramedullary injection revealed an uneven distribution of BrdU-positive cells in the bone marrow cavity, cartilage, and the subchondral area of the femoral head, as well as in trabecular bone, as evidenced by immunohistochemistry. Eight weeks after the injection, a further increased number of BrdU-positive cells could be observed in the bone marrow cavity and trabecular bone (Figure S4).

**Fig. 5:**
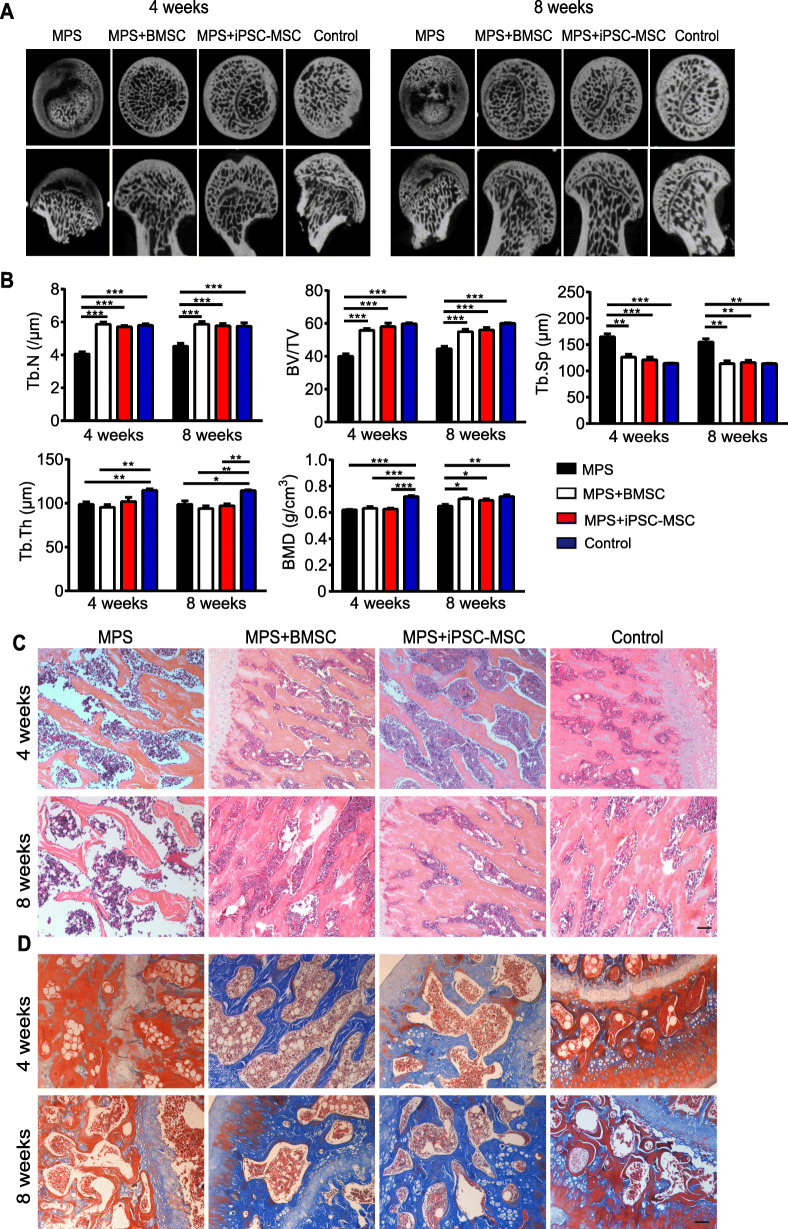
The osteogenesis-promoting effects of iPSC-MSCs on the SD rat model of MPS-induced ONFH. **a** Micro-CT imaging of the femoral head of each treatment group at 4 weeks and 8 weeks after the operation. **b** Micro-CT quantitative analysis of BMD, BV/TV, Tb.Sp, Tb.N, and Tb.Th (*n* = 5 per group). **c** Trabeculae and osteocytes analysis by HE staining for each treatment group at 4 weeks and 8 weeks after the operation. **d** Distinction of mature bone (red) and regenerated cartilage (blue) by Masson staining. Scale bar indicated 100 μm in all panels. All data are presented as mean ± SEM. Statistical differences were calculated using unpaired *t* test (*p < 0.05; **p < 0.01; ***p < 0.0001)

The bone tissues within the femoral head were analyzed qualitatively through Micro-CT scanning. A region of interest (ROI) was selected in the femoral head for observation, and statistical analysis was performed as described previously [47]. In the MPS group, in comparison to the normal control group, the necrosis area in the femoral head increased gradually with time, the trabecular bone in the femoral head was sparse and even completely disappeared in certain areas, and there was cystic degeneration in the subchondral area of the femoral heads. On the other hand, better structural integrity and distribution of the trabecular bone were observed in both the BMSC implant group and iPSC-MSC implant group (Fig. 5a). The quantitative analysis revealed a significant decrease in the BMD, BV/TV, Tb.Th, and Tb.N in the MPS group compared to the control group. Four weeks after implanting BMSCs or iPSC-MSCs, the decrease in these parameters induced by MPS was reversed (except for BMD and Tb.Th), while the change in BMD was reversed significantly (*p* < 0.01) 8 weeks after the implanting. In addition, Tb.Sp was remarkably increased in the MPS group compared to the control group, and this increase could be profoundly inhibited by implanting BMSCs or iPSC-MSCs (Fig. 5b).

The histological evidence based on HE staining revealed sparse trabecular bone, which even disappeared in certain areas, empty lacunae in the cavities of trabecular bone, and decreased osteocytes in the MPS group (Fig. 5c). Contrastingly, in both the BMSC-implant group and iPSC-MSC implant group, the trabecular bone structure was intact, only slight or no trabecular bone structure was replaced with necrotic tissue, and the number of osteocytes and chondrocyte had increased. Bone matrix is rich in collagen, which is closely associated with various bone diseases [48]. Masson staining allowed distinguishing between mature bone (red) and regenerated bone (blue). The results revealed a thin trabecular bone and a few regenerated bones or cartilage tissue in the MPS group. In BMSC and iPSC-MSC implant groups, massive regenerated bone tissues and the infiltration of a large number of the bone marrow cells in the medullary cavity were observed, indicating a remarkable promotion of osteogenesis in the necrosis area of the femoral head (Fig. 5d). In summary, both BMSC implanting and iPSC-MSC implanting could prevent bone loss and promote bone repair in the necrosis area of the femoral head.

### The iPSC-MSCs enhanced angiogenesis in the femoral head in steroid-induced ONFH rat model

**Fig. 6:**
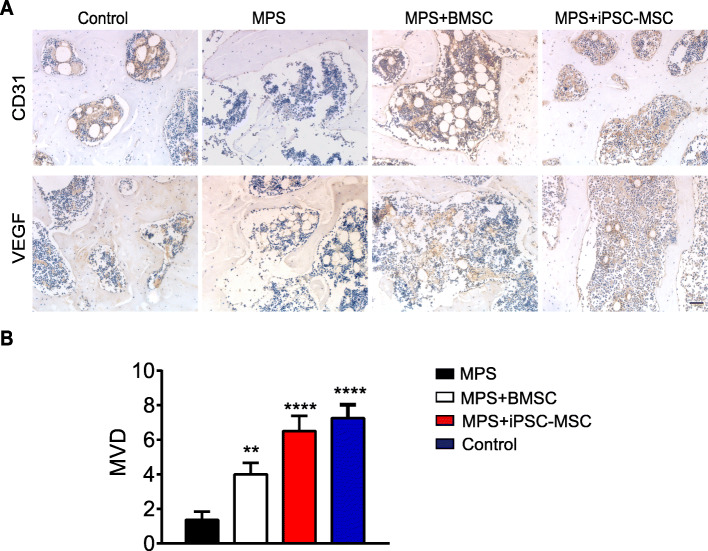
Angiogenesis in the femoral head. **a** Immunohistochemical analysis of the expression of VEGF and CD3. **b** Microvessel density (MVD) was calculated following IHC staining by counting single endothelial cells or clusters of endothelial cells. Data are presented as mean ± SEM. Statistical differences were calculated using unpaired t-test (**p < 0.01; ***p < 0.001; ****p < 0.0001). Scale bar indicated 50 μm

Since one of the main causes of ONFH is insufficient blood supply, angiogenesis in the femoral head was evaluated next by IHC staining. The results demonstrated that the expression of both VEGF and CD31 in the femoral head increased in the iPSC-MSC implant group in comparison to the MPS group (Fig. 6a). Microvessel density (MVD) was determined through IHC staining as described in a previous report [47]. In comparison to the MPS group, the iPSC-MSC implant group presented significantly increased (*p* < 0.0001) microvessels of the subchondral bone, which was higher than that in the BMSC implant group (*p* < 0.05) and similar to that in the control group (*p* = 0.54) (Fig. 6b). These findings indicated that iPSC-MSCs could effectively promote angiogenesis in the femoral head.

## Discussion

MSCs have demonstrated considerable prospects in the preclinical studies concerning ONFH treatment [21, 23, 49, 50]. However, the application of autologous or allogeneic BMSCs or MSCs derived from other tissues in large-scale clinical cellular therapy research encounters several challenges, including the limited proliferation capacity of MSCs, the variability of donor MSCs, and the costs associated with donor screening [51]. Therefore, an alternative standardized and inexhaustible source of MSCs exhibiting high activity, meant for cellular therapy, is required. Increasing evidence suggests that iPSCs can serve as a robust source of MSCs for clinical application [52, 53].

The emergence of the cellular reprogramming technology has enabled generating iPSCs from autologous histiocytic cells, which has resolved the issues of limited cell sources and immune rejection in cellular therapy, introducing novel strategies for regenerative medicine, disease models, and drug research and development. The selection of donor cells forms the basis of cellular reprogramming. Although it is possible to reprogram human skin fibroblasts into iPSCs [35], BMSCs were selected as donor cells for reprogramming in the present study, firstly because of the convenience of obtaining materials when the ONFH patients are undergoing total hip arthroplasty without secondary trauma, and secondly, because BMSCs are adult stem cells and may be easier to reprogram compared to terminally differentiated somatic cells. Furthermore, human iPSCs derived from donor cells have a donor-specific epigenetic memory, implying that the donor cell type could influence the differentiation potential of the derived iPSCs [54, 55]. Therefore, BMSC-derived iPSCs could be inclined to differentiate into MSCs. In this study, the potential for the application of iPSC-MSCs as an alternative cell source for the treatment of ONFH was investigated.

It was demonstrated that BMSCs are a suitable source for reprogramming, allowing the successful establishment of patient-specific iPSCs. The iPSCs generated in our study exhibited the characteristics of completely reprogrammed cells, including ESC-like colony morphology, alkaline phosphatase expression, and the expression of pluripotent markers such as Oct4, Sox2, Nanog, SSEA4, TRA-1–60, and TRA-1–81 (Fig. 1b–e). Moreover, in the generated iPSCs, the exogenous reprogramming genes were silenced, the expression of pluripotent genes was upregulated (data not shown), and multilineage differentiation potential in vivo was exhibited (Fig. 1f).

In the present study, a simple protocol of the differentiation of iPSCs into MSCs through EB formation was followed. The generated iPSC-MSCs fulfilled the basic criteria established by the Mesenchymal and Tissue Stem Cell Committee of the International Society for Cellular Therapy (ISCT) for use as the standard definition of human MSCs [45]. The results of the present study revealed that the generated iPSC-MSCs exhibited morphology, immunophenotype, and osteogenic and adipogenic differentiation ability similar to BMSCs (Fig. 2). In addition, the iPSC-MSCs exhibited better proliferative capability compared to the BMSCs, without forming teratomas. Since iPSC-MSCs are not immortal cells, they inevitably underwent senescence, similar to BMSCs under the same conditions, although senescence occurred at a later stage compared to that in BMSCs (Fig. 3). It was observed that, in the course of culture, normal BMSCs could be expanded to 8 passages, while ONFH-BMSCs could not be expanded to more than 5 passages. However, after reprogramming, the generated iPSC-MSCs could be expanded to 20 passages. According to a previous report, iPSC-MSCs underwent senescence and could not be expanded over 17 passages, similar to BMSCs [56]. These findings further indicate iPSCs as an attractive source for obtaining MSCs in huge numbers for therapeutic applications. Previous studies have revealed the implications of abnormal DNA methylation in the pathogenesis of severe bone and cartilage diseases, such as primary osteoarthritis (OA) [57]. Although the studies focusing on DNA methylation profiling of MSCs in ONFH are scarce, there is sufficient evidence in support of DNA methylation having an important role in the pathogenesis of ONFH. In the present study, ONFH-specific epigenetic differences could be eliminated with reprogramming without requiring reconstruction in iPSC-MSCs. Moreover, iPSC-MSCs exhibited a methylation pattern that resembled the methylation pattern of normal BMSCs rather than the parent cells (Fig. 4), suggesting that reprogramming could reverse the abnormal DNA methylation in ONFH-BMSCs, which might serve as a key factor for recovering the activity of ONFH-BMSCs.

To facilitate tracking of the transplanted MSCs, the MSCs were labeled in vitro before transplantation. GFP labeling is an easy-to-observe widely-used labeling method [58]. However, GFP labeling requires a virus vector, is inefficient, and also affects the cytoactivity. Therefore, in this study, BrdU labeling was applied as described in a previous report [15]. Micro-CT scanning revealed that the transplantation of iPSC-MSCs and BMSCs could effectively improve the BMD, Tb.N, and BV/TV, while there were no significant changes in the Tb.Th (Fig. 5). The reason for this could be a limited experimental duration. Although the new trabecular bones were dense, they were not sufficiently mature. Further studies with extended periods of observation may be able to identify significant changes in Tb.Th as well.

## Conclusions

In summary, the present study is a pioneer in demonstrating the equivalence of iPSC-MSCs and BMSCs concerning the treatment of steroid-induced ONFH in SD rats. In addition, it was demonstrated that the abnormal biological function and DNA methylation patterns of BMSCs in ONFH patients could be reversed by reprogramming these cells into iPSC-MSCs. The generated iPSC-MSCs exhibited morphology, immunophenotype, and in vitro differentiation potential analogous to normal BMSCs while exhibiting higher proliferation rates. Furthermore, iPSC-MSC implanting could effectively promote bone repair and angiogenesis in the necrosis area of the femoral head. Generating reprogrammed iPSC-MSCs represents an effective approach to provide a stable and reliable source of therapeutic cells, which could provide a novel direction of standardized MSCs to the treatment of ONFH in the future.

## Supplementary Information

**Additional file 1: Figure S1.** Flow cytometric analysis for the cell surface markers of BMSCs at passage 3. **Figure S2.** Karyotype analysis. The karyotype of iPSC was normal (46, XY) after 37 passages. **Figure S3.** The immunogenicity and immunoregulatory function of MSCs. Flow cytometric analysis for immune-related surface molecules (CD80/CD86) of BMSCs (**a**) and iPSC-MSC (**b**). **c** Activity of iPSC-MSCs and BMSCs on the proliferation of stimulated T cells. **Figure S4.** Immunohistochemical staining of BrdU at the 4 weeks and 8 weeks after cell implantation. Scale bar indicated 50 μm. **Figure S5.** Gene expression of osteogenic gene Collagen type I and ALP after two weeks of osteogenic induction. For each gene, expression levels were normalized to the control group, *n* = 4 per group. All data are presented as mean ± SEM. One-way ANOVA with Tukey’s adjustment (*, *p* < 0.05; **, *p* < 0.01; ***, *p* < 0.0001). **Table S1.** Clinical characteristics of recruited patients. **Table S2.** Sequence of RT-qPCR primers.

## Data Availability

The data that support the findings of this study are available from the corresponding author on reasonable request.

## Abbreviations

iPSCs: Induced pluripotent stem cells
ESCs: Embryonic stem cells
ONFH: Osteonecrosis of the femoral head
BMSCs: Bone marrow mesenchymal stem cells
ONFH-BMSCs: Bone marrow mesenchymal stem cell-derived from osteonecrosis of the femoral head patient
iPSC-MSCs: Mesenchymal stem cells derived from iPSCs
OSX: Osterix
SD: Sprague-Dawley
MPS: Methylprednisolone
BMD: Bone mineral density
Tb.Th: Trabecular thickness
Tb.Sp: Trabecular separation
BV/TV: Bone volume per tissue volume
Tb.N: Trabecular number
MVD: Microvessel density
