# Supplementary material for: Reprogrammed mesenchymal stem cells derived from iPSCs promote bone repair in steroid-associated osteonecrosis of the femoral head
Source: Stem Cell Res Ther. 2021 Mar 12;12:175. doi: 10.1186/s13287-021-02249-1 (PMC7953570; doi:10.1186/s13287-021-02249-1)
Supplement: Supplementary file 1 — Additional file 1: Figure S1. Flow cytometric analysis for the cell surface markers of BMSCs at passage 3. Figure S2. Karyotype analysis. The karyotype of iPSC was normal (46, XY) after 37 passages. Figure S3. The immunogenicity and immunoregulatory function of MSCs. Flow cytometric analysis for immune-related surface molecules (CD80/CD86) of BMSCs (a) and iPSC-MSC (b). c Activity of iPSC-MSCs and BMSCs on the proliferation of stimulated T cells. Figure S4. Immunohistochemical staining of BrdU at the 4 weeks and 8 weeks after cell implantation. Scale bar indicated 50 μm. Figure S5. Gene expression of osteogenic gene Collagen type I and ALP after two weeks of osteogenic induction. For each gene, expression levels were normalized to the control group, n = 4 per group. All data are presented as mean ± SEM. One-way ANOVA with Tukey’s adjustment (*, p < 0.05; **, p < 0.01; ***, p < 0.0001). Table S1. Clinical characteristics of recruited patients. Table S2. Sequence of RT-qPCR primers. [file 13287_2021_2249_MOESM1_ESM.pdf]

Supplementary information

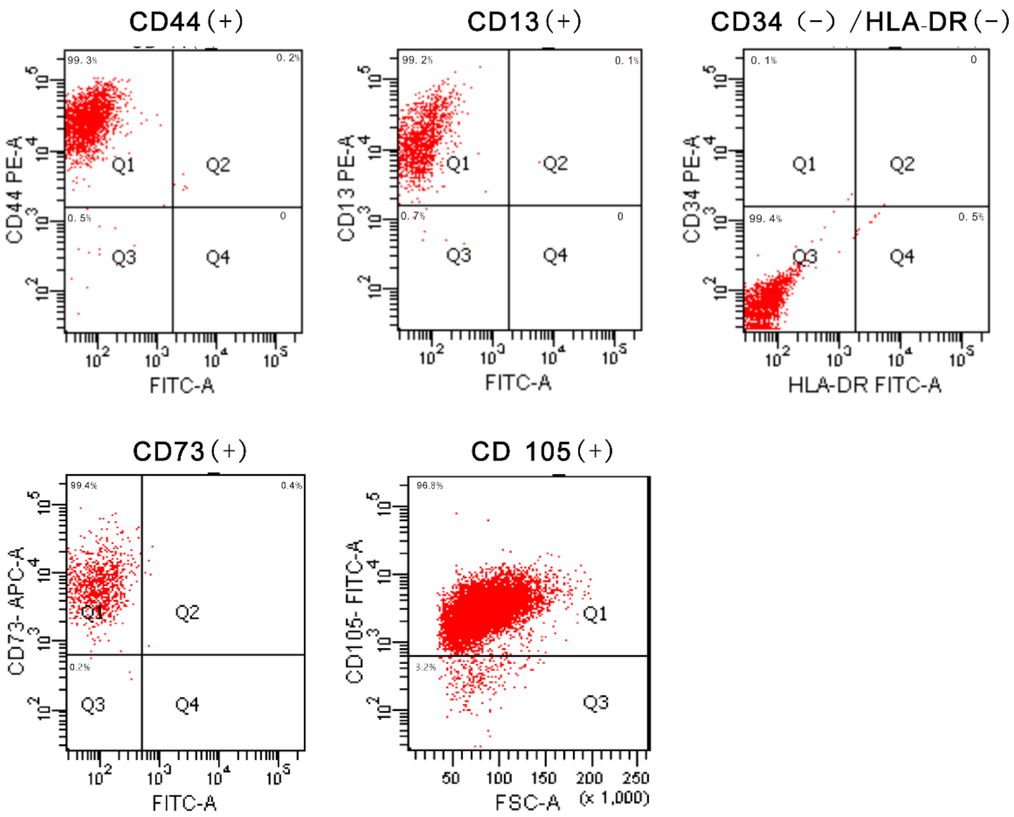

Figure S1. Flow cytometric analysis for the cell surface markers of BMSCs at passage 3.

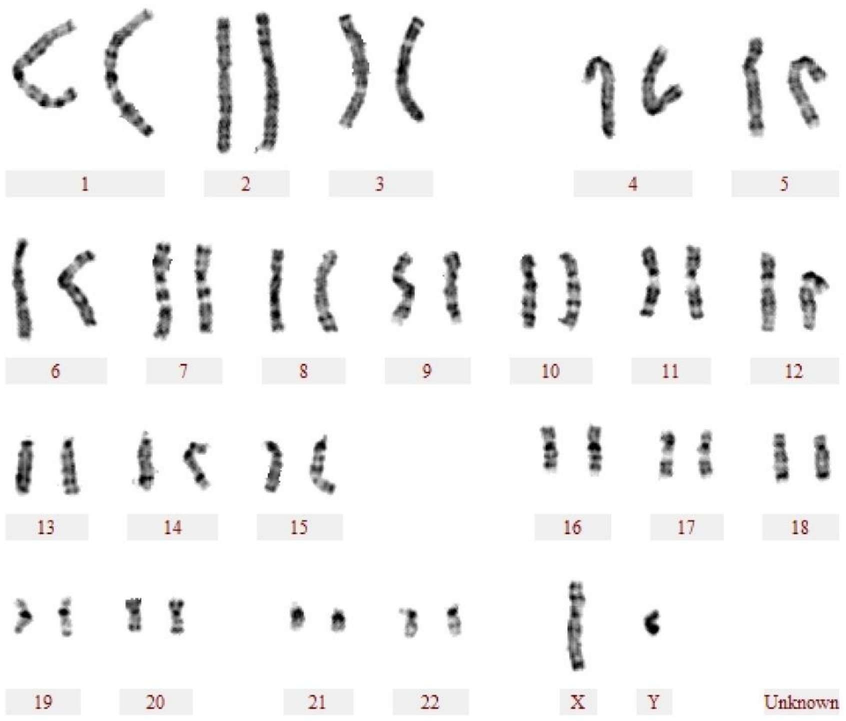

Figure S2. Karyotype analysis. The karyotype of iPSC was normal (46, XY) after 37 passages.

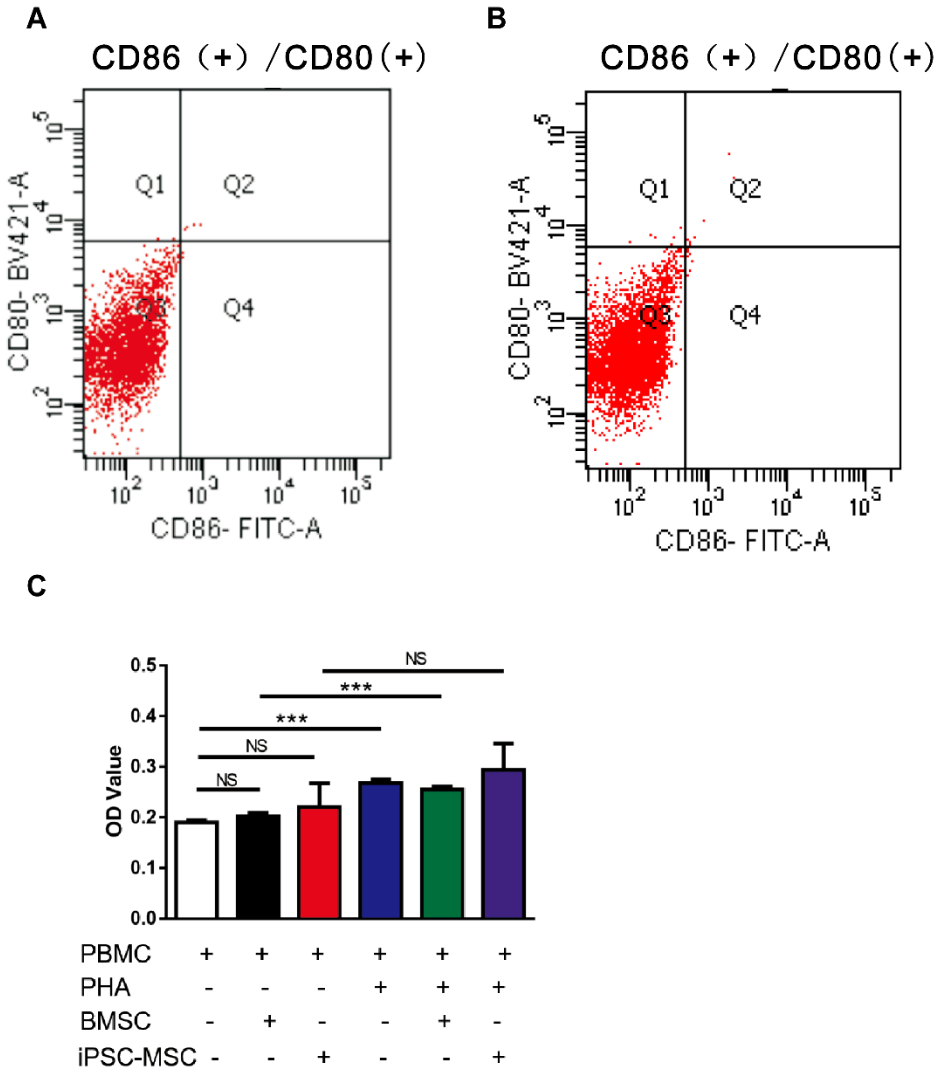

Figure S3. The immunogenicity and immunoregulatory function of MSCs. Flow cytometric analysis for immune-related surface molecules (CD80/CD86) of BMSCs (**a**) and iPSC-MSC (**b**). **c** Activity of iPSC-MSCs and BMSCs on the proliferation of stimulated T cells.

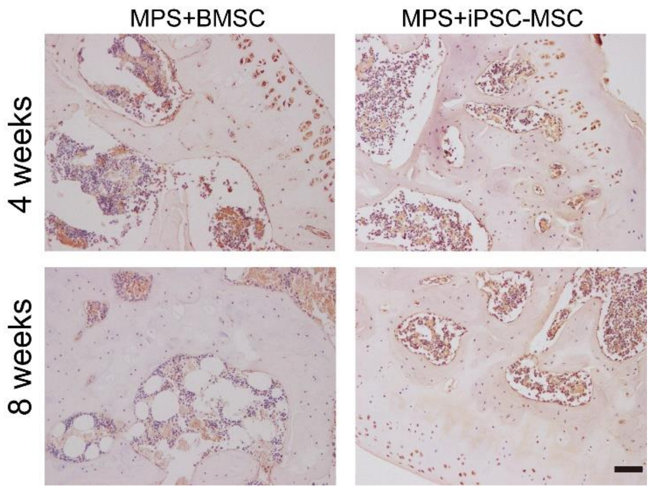

Figure S4. Immunohistochemical staining of BrdU at the 4 weeks and 8 weeks after cell implantation. Scale bar indicated 50 μm.

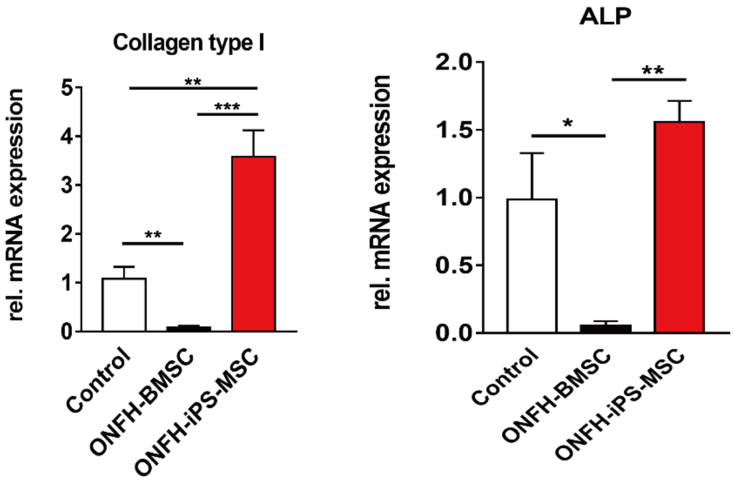

Figure S5. Gene expression of osteogenic gene Collagen type I and ALP after two weeks of osteogenic induction. For each gene, expression levels were normalized to the control group, n = 4 per group. All data are presented as mean ± SEM. One-way ANOVA with Tukey's adjustment (*, $p < 0.05$; **, $p < 0.01$; ***, $p < 0.001$).

Table S1

Clinical characteristics of recruited patients

Characteristics	Control (n=12)	ONFH (n=16)
Age (year)	59.3±11.9	45.1±14.6
Gender (male/female)	8/4	9/7
Body mass index (kg/m^2)	20.2 ± 2.4	24.1 ± 2.9
Hypertension	1 (8.3%)	3 (18.8%)
Hyperlipidemia	1 (8.3%)	4 (25%)
Smoker	8 (66.7%)	10 (62.5%)
Alcoholism	6 (50%)	6 (50%)
GC medication	0	4 (25%)

Control: femoral neck fractures; ONFH: osteonecrosis of the femoral head; GC: glucocorticoid;

Table S2

Sequence of RT-qPCR primers

Genes		Primer sequence (5'-3')	Product size (bp)
GAPDH	Forward	GAGTCAACGGATTTGGTCGT	185
	Reverse	GACAAGCTTCCCGTTCTCAG	
Runx 2	Forward	CGCATTCCTCATCCCAGTAT	118
	Reverse	GCCTGGGGTCTGTAATCTGA	
OSX	Forward	ACCCAACATCAAGCAGAACC	120
	Reverse	TTCAACTTCCCAGGCTCACT	
PPARγ	Forward	CGAGAAGGAGAAGCTGTTGG	122
	Reverse	TCAGCGGGAAGGACTTTATG	
